# Effects of lower-limb active resistance exercise on mobility, physical function, knee strength and pain intensity in patients with total knee arthroplasty: a systematic review and meta-analysis

**DOI:** 10.1186/s12891-024-07845-9

**Published:** 2024-09-12

**Authors:** Guo Wei, Zhenghui Shang, Yupeng Li, Yu Wu, Li Zhang

**Affiliations:** 1 Department of Orthopedics, The First College of Clinical Medical Science, China Three Gorges University, Yichang Central People’s Hospital, Yichang City, Hubei Province China; 2 Department of Critical Care Medicine, The First College of Clinical Medical Science, China Three Gorges University, Yichang Central People’s Hospital, Yichang City, Hubei Province China

**Keywords:** Active resistance exercise, Total knee arthroplasty, Rehabilitation, Mobility, Physical function

## Abstract

**Background:**

Total knee arthroplasty (TKA) successfully alleviates pain from knee osteoarthritis, but muscle strength and function are reduced for a long period postoperatively. Postoperative active resistance exercise may play a relevant role.

**Purpose:**

To systematically evaluate effects of lower-limb active resistance exercise (ARE) on mobility, physical function, muscle strength and pain intensity in patients with TKA.

**Methods:**

A search was conducted in PubMed, EMBASE, and Cochrane Library databases from inception to September 2023. Only randomized controlled trials (RCTs) that compared the effects of ARE and no intervention or other rehabilitation program without PRE were included. The outcome variables were mobility (Maximal walking speed [MWS]/6-Minute Walk Test[6MWT]), physical function (Stair Climb Test [SCT]/Timed Up and Go [TUG]), knee extension/ flexion power(KEP/KFP), joint range of motion (ROM) and pain. Standardized Mean Differences (SMD) or Mean Differences (MD) and 95% confidence intervals (CI) were calculated and combined in meta-analyses. The Cochrane Collaboration’s Handbook were used for the methodological quality assessments. GRADE was used to assess the quality of evidence. The meta-analysis was performed using the RevMan 5.4 software.

**Results:**

A total of 14 randomized controlled trials, involving 880 patients, were finally included. The lower-limb ARE exhibited significantly greater improvement in MWS (MD 0.13, 95%CI 0.08–0.18, *P* < 0.00001), TUG(MD -0.92, 95%CI -1.55– -0.28, *P* = 0.005), KEP (SMD 0.58, 95%CI 0.20–0.96, *P* = 0.003), KFP (SMD 0.38, 95%CI 0.13–0.63, *P* = 0.003), ROM-flexion (MD 2.74, 95%CI 1.82–3.67, *P* < 0.00001) and VAS (MD − 4.65, 95% CI − 7.86– -1.44, *p* = 0.005) compared to conventional exercise(CE) immediately post-intervention. However, there were no statistically significant differences between both groups in regard to 6MWT (MD 7.98, 95%CI -4.60–20.56, *P* = 0.21), SCT (MD -0.79, 95%CI -1.69–0.10, *P* = 0.08) and ROM-extension (MD -0.60, 95%CI -1.23–0.03, *P* = 0.06).

**Conclusions:**

According to the results of meta-analysis, patients undergoing TKA who receive the lower extremity ARE show better clinical effects in terms of pain relief, strength recovery and knee ROM. Simultaneously, it may be beneficial to improve mobility and physical function of patients after TKA.

**Supplementary Information:**

The online version contains supplementary material available at 10.1186/s12891-024-07845-9.

Osteoarthritis of the knee is a common chronic and degenerative disease that can cause joint stiffness, pain, swelling and loss of mobility [1]. Total knee arthroplasty (TKA) is an effective treatment to alleviate pain and disability for end-stage knee osteoarthritis [2]. Although TKA reliably relieves pain and improves self-reported function, patients continue to exhibit the significant reduction in quadriceps strength, hamstring muscle strength and functional performance [3, 4]. Additionally, the loss of lower extremity muscle strength is associated with functional decline [5–7]. In a systematic review of controlled trials, Pozzi et al. [8] indicated that lower extremity strength, particularly quadriceps strength, was highly related to functional performance and should certainly be included in any intervention that targets muscular impairments after TKA. Therefore, postoperative rehabilitation is particularly crucial for the achievement of good joint function and high patient satisfaction.

Effort has been devoted to enhancing postoperative rehabilitation, with various interventions ranging from physiotherapy, active and passive exercises, to maximize recovery and reduce post-operative complications [8–10]. The conventional rehabilitation strategies commonly focus on functional exercises with no or low resistance. Continuous passive motion (CPM) is often used as a routine form of rehabilitation to improve knee joint range of motion (ROM) during postoperative rehabilitation. However, its effects on physical function of TKA patients are controversially discussed, just as a meta-analysis by Yang X et al. pointed out that neither the ROM nor the functional outcomes could be improved by CPM therapy [11]. In 2018, Schulz M, et al. proposed that the controlled active motion appeared to produce better improvement of flexion, pain and quality of life in comparison to CPM [12]. Besides, some recent related articles showed that the lower-extremity active resistance exercise (ARE) program provided additional benefits over the conventional exercise(CE) to patients with TKA, such as reducing pain, increasing muscle strength and range of motion, and improving physical function and gait [13–18]. Considering that the loss of muscle strength was related to functional impairments and biomechanical asymmetries [19, 20], lower-limb ARE should be conducted shortly after surgery.

The effect of exercise to increase lower extremity power may be important for postoperative functional rehabilitation. Most pilot randomized controlled trials (RCTs)had shown that all kinds of ARE had a superior effect on knee functional recovery, when compared to CE [12–16, 21–28]. Nonetheless, previous studies had advocated that ARE seems to provide similar improvement of functional rehabilitation in comparison to CE [29, 30]. Moreover, the clinical effects of ARE or CE have not been researched in a larger scale by a randomized controlled study. This evidence-based study, therefore, set out to identify the effectiveness of the lower extremity ARE on the available clinical outcomes, such as mobility, physical function, knee extension/flexion power, ROM and pain, based on evidence from published high-quality RCTs.

## Methods

### Protocol and registration

The review was conducted and reported according to the Preferred Reporting Items for Systematic Review and Meta-Analysis statement [31]. It was registered on the PROSPERO (CRD42024498809).

### Search strategy

A comprehensive search strategy involved a literature search of published articles in the English language through the medical databases of PubMed, EMBASE and Cochrane’s Library up to September 2023. The following key words were used for the database research: “total knee arthroplasty”, “total knee replacement”, “rehabilitation”, “strengthening”, “resistance”, “training”, “exercise”, “active” and “progressive”. Additionally, the reference lists of the included studies were also assessed for eligible articles to expand search results. The detailed search strategy could be found in supplementary file 1. Two investigators (WG and SZH) independently searched all the titles and abstracts related to inclusion criteria. The potentially relevant articles underwent full-text retrieval independently and repeatedly by two independent investigators (WG and SZH). Any divergence was resolved through discussion or consultation with a third author (LYP).

### Inclusion criteria and exclusion criteria

The screening of the potential eligible records was based on the following inclusion criteria: 1) participants (P): patients operated with TKA; 2) intervention (I): active resistance exercise of the lower limbs. There were no restrictions on the manner, frequency, duration, and intensity of resistance exercise; 3) comparison (C): no intervention or other rehabilitation programs without ARE, such as CPM, a home-based functional non-ARE exercise, or functional exercises with low resistance(body weight exercises); 4) outcomes (O): Maximal walking speed (MWS), 6-Minute Walk Test(6MWT), Stair Climb Test(SCT), Timed Up and Go(TUG), knee extension power(KEP), knee flexion power(KFP), Knee range of motion(KROM) flexion or extension and Visual Analogue Scale(VAS); 5) study (S): only RCTs, which were published in English, were eligible for the inclusion in this study.The exclusion criteria were as follows: 1) review articles, abstracts, letters and case report; 2) absence of any available outcome; 3) studies reporting measurement data with median values or not providing measures of dispersion in the form of standard deviation;4) ongoing clinical trials not published in full.

### Data extraction

Two investigators independently extracted data from the studies meeting the inclusion and exclusion criteria. Any disagreements were resolved through discussion or consultation with a third reviewer.The following data were independently extracted from each eligible study: first author, publication year, study design, number of patients, demographic information, follow-up durations, intervention characteristics and all the outcome parameters which consisted of the power of mobility, function, knee extension/ flexion power, ROM, and pain. The primary outcomes of the study were mobility, knee function and knee strength, presented as MWS, 6MWT, TUG, SCT and knee extension/ flexion power among included studies [32, 33]. The secondary outcomes included ROM and pain derived from the pain visual analogue scale (VAS).

### Risk of bias assessment and assessment of certainty of evidence

Two investigators independently evaluated the Risk of bias of the included studies and the quality of evidence. If there were any disagreements to reach the final assessment, a third author was consulted. The qualities of the included studies were assessed strictly according to the Cochrane risk of bias assessment criteria (Cochrane RoB 2 tool) [34]. To assess confidence in the combined estimates of effect, we applied the GRADE (Grading of Recommendations Assessment, Development and Evaluation) approach using the following criteria: risk of bias, consistency, directness, precision, and reporting bias [35]. We considered the five standard domains for downgrading evidence in GRADE to inform an overall assessment of certainty for each outcome, which was judged to be high, moderate, low and very low.

### Statistical analysis

Meta-analysis was performed separately using Review Manager 5.4 software, when there were available data that could be combined. Our outcome was the score at the last follow-up period rather than the change in score as this maximized the number of comparable studies. For continuous results, the mean and standard deviation (SD) were used to calculate the mean difference (MD)or standardized mean difference (SMD) and 95% confidence interval (CI). MD was used when the data units are the same, and SMD was used when the data units were different. When SDs were not provided, the authors calculated them for meta-analysis purposes. The heterogeneity between studies was tested by using the I^2^ statistic and the χ2 test. If I^2^ statistic < 50% and *P* > 0.05, the heterogeneity was suggested to be small, and a Fixed Effect model was used. If I^2^ statistic > 50%or *P* < 0.05, the data was considered to have substantial heterogeneity and a random-effect model was selected. When heterogeneity existed, a sensitivity analysis was performed to evaluate the influence of the individual study on the pooled results by omitting every single study per iteration. If it was possible to check various factors affecting ARE, a subgroup analysis might be performed according to the time point and duration for starting ARE after surgery. Publication bias tests were performed for those outcome indicators with included studies more than 10. A value of *P* < 0.05 was regarded as statistically significant.

## Results

### Literature search results and study characteristics

**Fig. 1:**
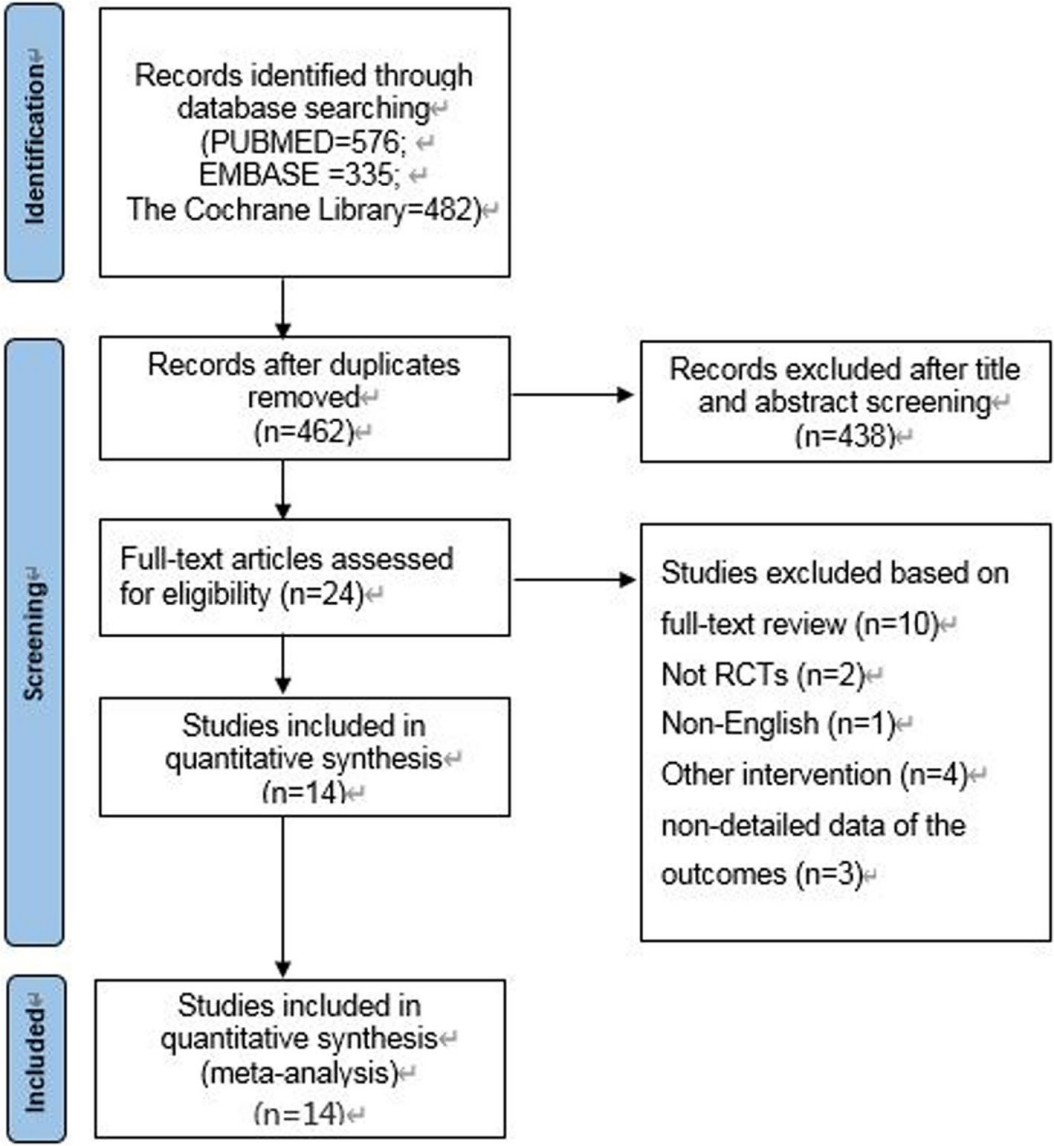
Flow diagram of the literature search

The process of literature selection was summarized in Fig. 1. The baseline information of these studies is listed in Table 1. The primary literature search identified 1393 potentially relevant titles from the databases. After discarding the duplicate studies and reading the titles and abstracts of the articles, 1369 publications were excluded. The remaining articles were further assessed for eligibility based on the full text articles. Table 2 presented a detailed list of excluded studies. Eventually, 14 RCTs [12, 13, 15, 16, 21–30]were identified for data collection and critical assessment. All included studies consisted of a intervention group and a control(CON) training group. The 14 studies involved 443 participants who received ARE and 437 who received a CON intervention.

**Table 1:** Characteristics of the included RCTs

Study (Year)	Study design	Participants		intervention		Outcomes
		Intervention	Control	Intervention	Control	
Valtonen A et al. 2010 [21]	RCT	26 patients; 61.5% female; Mean Age 66.2 ± 6.3; Follow-up 24–84 weeks; Exercise duration 12 weeks	24 patients; 58.3% female; Mean Age 65.7 ± 6.0; Follow-up 24–84 weeks; Exercise duration 12 weeks	Twelve-week progressive aquatic resistance training; (1) knee extension-flexion movement in a sitting position,(2) hip abduction–adduction with extended knee in a standing position, (3) hip extension-flexion with extended knee in a standing position, (4) knee extension-flexion in a standing position, and (5) step-squat backward from the aqua aerobic step board. The progression of the exercise program was ensured by using resistance boots of different sizes and by varying the amount and duration of sets	Not receive any intervention. Participants were encouraged to continue their lives as usual and maintain their habitual level of physical activity during the trial	MWS VAS KEP KFP
Christiansen CL et al. 2015 [30]	RCT	13 patients; 46.2% female; Mean Age 68.2 ± 8.6; Follow-up 26 weeks; Exercise duration 6 weeks	13 patients; 53.8% female; Mean Age 66.6 ± 8.1; Follow-up 26 weeks; Exercise duration 6 weeks	The Nintendo Wii Fit Plus game and associated Wii Balance Board were utilized to supervise Weight-bearing Biofeedback Progression Tasks, including activities of bilateral stance, unilateral stance, sit to stand, and lunging	Patients in both groups were contacted on a weekly basis to remind them to complete the home exercise program	MWS KEP
Husby VS et al. 2018 [28]	RCT	21 patients; 52.4% female; Mean Age 61(46–72); Follow-up 48 weeks; Exercise duration 8 weeks	20 patients; 60% female; Mean Age 63(45–73); Follow-up 48 weeks; Exercise duration 8 weeks	Strength training consisted of 4 series of 5 repetitions with a load corresponding to 80–90% of 1RM, with focus on high velocity in the concentric part of the movement and with resting periods of 1–2 min. When participants were able to perform 6RM, the load was increased by 5 kg for leg presses and by 0.5–1 kg for knee extensions. All training sessions lasted 30 min and were supervised by an exercise physiologist /physiotherapist	received a weekly phone call from the project leader to investigate the overall health of the individual participant, including the progress of home exercise log	KEP KFP 6MWT
Kelly MA et al. 2016 [25]	RCT	19 patients; 68.4% female; Mean Age 70 ± 6; Follow-up 8 weeks; Exercise duration 7 weeks	19 patients; 57.9% female; Mean Age 73 ± 8; Follow-up 8 weeks; Exercise duration 7 weeks	Standardized exercise sessions were performed by the groups. Curbs and stairs were performed as fast as possible while maintaining proper form. Open-chain resistive exercises were completed with a concentric contraction in 1 s or less. Walking forward, step up/down, and ascending and descending a flight of stairs were performed as fast as and was comfortable without any increased gait deviation	Standardized exercise sessions were performed by the groups. Curbs and stairs were performed at their comfortable preferred speed. Open-chain resistive exercises were performed with a concentric contraction in 2 s. Walking forward, step up/down, and ascending and descending a flight of stairs were performed by the LV group at their preferred speed	6MWT TUG SCT VAS Gait velocity
Jakobsen TL et al. 2014 [29]	RCT	35 patients; 40% female; Mean Age 66(56–73); Follow-up 26 weeks; Exercise duration 7 weeks	37 patients; 43.2% female; Mean Age 63(57–68); Follow-up 26 weeks; Exercise duration 7 weeks	Warming up for 5 min: the patients used both legs as soon as they were able to flex their operated knee 95°; The range of motion exercise with 3 min was performed on a stationary bike with adjustable seat height to improve knee joint range of motion of the operated leg; Stretching exercises of the knee extensors, knee flexors, and calf muscles of the operated leg were 4 min, Progressive strength training for 15 min of knee extension and leg press, with training load; And then functional training (10 min), balance training (5 min), and icing and evaluation (5 min)	The identical physical rehabilitation with PST group, except that the PST component (15 min)was replaced with warming up (5 min), knee range of motion exercise (3 min), knee extensor stretches (2 min), and 1-legged balance exercise at multiple angles (5 min)	6MWT KEP KFP ROM
Bade MJ et al. 2017 [25]	RCT	84 patients; 46.4% female; Mean Age 63 ± 8; Follow-up 48 weeks; Exercise duration 11 weeks	78 patients; 43.6% female; Mean Age 64 ± 7; Follow-up 48 weeks; Exercise duration 11 weeks	It was a high-intensity, progression-based, rehabilitation program based on prior research and consisted of several domains: a warm-up; PRE targeting the ankle plantar flexors, quadriceps, hamstrings, and hip abductors, adductors, extensors and flexors; bilateral and unilateral weight-bearing functional exercises; balance exercises; agility exercises; and activity prescription	It was a time-based rehabilitation program and key differences from the HI program were: 1) an initial focus on isometric and ROM exercise for the first 4 weeks 2) a slower transition to weight-bearing exercises 3) less progression in difficulty of weight-bearing exercises 4) no resistance beyond body weight or elastic bands 5) restricted activity outside of ADLs for the first 4 weeks gradually building to 30 min by the end of therapy	SCT TUG 6MWT ROM
Heikkilä A et al. 2017 [26]	RCT	53 patients; 56.6% female; Mean Age 69 ± 8; Follow-up 56 weeks; Exercise duration 48 weeks	55 patients; 65.5% female; Mean Age 64 ± 9; Follow-up 56 weeks; Exercise duration 48 weeks	The same written exercise program. The individual guidance was provided at 2 months after TKA. From 2 months to 6 weeks, the program consisted of isometric strengthening exercises for the quadriceps and hamstring muscles at multiple knee joint angels performed in a sitting position. In the functional exercises, the participant’s body weight was used as a resistance. At 3 months, the patients were given new home exercise, included squats, hack squats and step exercise. At 4 months, the previously used exercises were increased. In the squat and hack squat exercises, the patients were instructed to increase the knee angle and the load on the operated side	With one week of hospitalization, the written exercise program was received, which included active and passive knee range of motion exercises knee flexor and extensor exercises and hip abductor and extensor exercises in the standing position using the weight of the extremity as a resistance. They didn't receive any additional guidance after discharge from hospital	KEP KFP VAS MWS
Hardt S et al. 2018 [27]		22 patients; No gender reported; Mean Age 63.8 ± 8; Follow-up 1 weeks; Exercise duration 1 weeks	25 patients; No gender reported; Mean Age 67.6 ± 10.2; Follow-up 1 weeks; Exercise duration 1 weeks	The same physiotherapy. In addition, the training group postoperatively performed an app-based feedback-controlled active progressive resistance training program multiple times daily	The same physiotherapy for both groups included active and passive knee mobilisation, gait training, assisted walking with crutches, strength exercises, stair climbing, manual phatic drainage, and cryotherapy three times daily with ice packs	ROM TUG 10MWT
Jacksteit R et al. 2021 [16]	RCT	22 patients; 59.1% female; Mean Age 67.36 ± 9.61; Follow-up 12 weeks; Exercise duration 3 weeks	22 patients; 63.6% female; Mean Age 68.45 ± 9.07; Follow-up 12 weeks; Exercise duration 3 weeks	In addition to the same Inpatient and outpatient rehabilitation programmes, they received a low-load resistance training of the operated leg three times daily for ∼30 min with a continuous active motion (CAM) devices. They performed active knee extensions and flexions through a controlled ROM	In addition to the same Inpatient and outpatient rehabilitation programmes, they received three Continuous passive motion (CPM) interventions per day for 30 min each	ROM VAS TUG SCT
Schulz M et al. 2018 [12]	RCT	25 patients; 48% female; Mean Age 69 ± 8; Follow-up 5 weeks; Exercise duration 5 weeks	25 patients; 56% female; Mean Age 71 ± 8; Follow-up 5 weeks; Exercise duration 5 weeks	The controlled active motion was conducted with the motion device. The detailed CAM protocol was not reported. Both groups received he same additional physiotherapeutic program	The continuous passive motion was conducted with the motion device. The detailed protocol was also not reported	VAS ROM
Mau-Moeller A et al. 2014 [22]	RCT	19 patients; 36.8% female; Mean Age 68.8 ± 8; Follow-up 12 weeks; Exercise duration 3 weeks	19 patients; 47.4% female; Mean Age 67.1 ± 8.8; Follow-up 12 weeks; Exercise duration 3 weeks	The same standardized in-hospital physiotherapy. The participants in the Intervention group performed active knee flexions and extensions in a sling while lying in a supine position. Exercise progression was achieved by asking the patients to gradually increase the range of motion as tolerated	The continuous passive motion protocol was started with 0° to the maximum tolerated flexion at the highest, adjustable speed. ROM was increased daily depending on tolerance	VAS ROM
Eymir M et al. 2021 [15]	RCT	58 patients; 84.5% female; Mean Age 68.9 ± 8.9; Follow-up 12 weeks; Exercise duration to discharge	55 patients; 90.5% female; Mean Age 68.9 ± 8.3; Follow-up 12 weeks; Exercise duration to discharge	The same standard physiotherapy was started on the postoperative day 1. The intervention group received active heel-slide exercise. The patient gently flexed the knee to a tolerable angle with heel slide, and to hold the knee at the achieved flexion angle for 5 s. After this, the patient extended the knee joint with heel slide to 0° of extension. One cycle of this active flexion–extension lasted about 30 s., and the patients performed 60 repetitions in 3 sets, with a 1-min break after each set	Except the same standard hysiotherapy, the patient laid in a supine position with the operated leg in the continuous passive motion machine. The CPM angle was started with 0° to the maximum tolerated flexion angle of the patient on POD1, and was increased pending on the tolerance of the patient per day	ROM TUG 10MWT
Do K et al. 2020 [13]	RCT	19/20 patients; 84.2/85% female; Mean Age 72.84 ± 7.03/72.50 ± 4.73; Follow-up 12 weeks; Exercise duration 4 weeks	16 patients; 81.3% female; Mean Age 73.13; Follow-up 12 weeks; Exercise duration 4 weeks	The hip group exercise programs included warm up, Supine extension bridge, Sideway walking, Standing hip adduction and Clamshell (Hip external rotation) with thera-band. The quadriceps group exercise programs included warm up, seated knee extension, supine straight leg raise and quarter wall squat with thera-band	Patients were randomly divided into three groups: hip, quadriceps, and control. The control group performed Active Range of Motion of the knees	ROM TUG 6MWT Gait velocity
Bily W et al. 2016 [23]	RCT	26 patients; 69% female; Mean Age 68.3 ± 6.7; Follow-up 12 weeks; Exercise duration form7 to 12 weeks	29 patients; 65.4% female; Mean Age 64.9 ± 6.0; Follow-up 12 weeks; Exercise duration form7 to 12 weeks	Both groups underwent a standard inpatient and outpatient rehabilitation program for six weeks. Six weeks after TKA, the intervention group started the strength training program on a computer-controlled, linear motor-powered leg-press machine. Patients underwent one-legged isokinetic concentric and eccentric leg-press training in sitting position to train hip extensor, knee flexor and extensor muscles in both legs	Six weeks after TKA, The control group's patients were treated in standardised one-on-one sessions with the main focus on functional improvements. Each session consisted of ergometer cycling, manual therapy and soft tissue techniques to improve scar and joint mobility, ROM-exercises, isometric and dynamic strengthening exercises, and gait-retraining exercises	VAS ROM TUG SCT KEP

*RCT* Randomized controlled trial, *MWS* Maximal walking speed, *HWS* Habitual walking speed, *KEP* Knee extension power, *KFP* Knee flexion power, *6MWT* 6-Minute Walk Test, *SCT* Stair Climb Test, *TUG* Timed Up and Go, *VAS* Visual Analogue Scale, *ROM* Range of motion

**Table 2:** Studies excluded from the review

Reason for exclusion	Study
Not a randomized clinical trial (*n* = 2)	Hsu et al. [9], Pozzi et al. [14]
Not-English (*n* = 1)	Kang et al. [17]
Other intervention (*n* = 4)	Bäcker et al. [36], Bade et al. [37], Wu et al. [38], Blasco et al. [39]
Not-detailed data of the outcomes (*n* = 3)	Schache et al. [40], Petterson et al. [20], Sanzo et al. [18]

### Assessment of risk of bias and certainty in the body of evidence

**Fig. 2:**
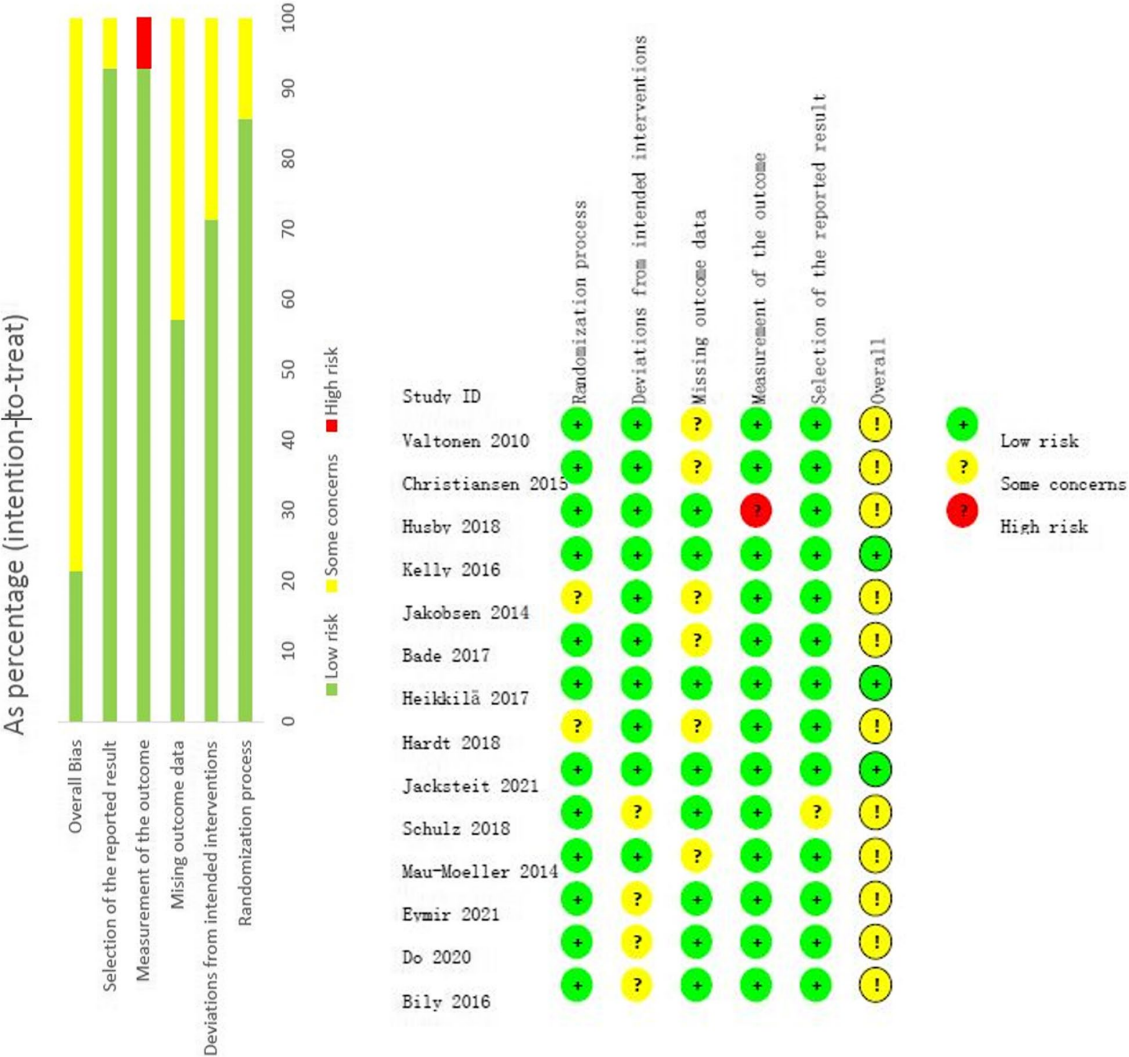
Risk of bias graph and summary

The quality of the included studies was assessed according to the cochrane risk of bias assessment criteria (Cochrane RoB 2 tool) (supplementary file 2) and the results were shown in Fig. 2. In general, most of the studies (79%;*n* = 11) were judged as ‘some concern” whereas three studies (21%) were considered to have “low risk of bias”. The quality of evidence from our meta-analysis was low(supplementary file 3). We downgraded the evidence because of risk of bias in studies, heterogeneity and imprecision.

### Primary outcomes

#### Mobility (MWS and 6MWT)

**Fig. 3:**
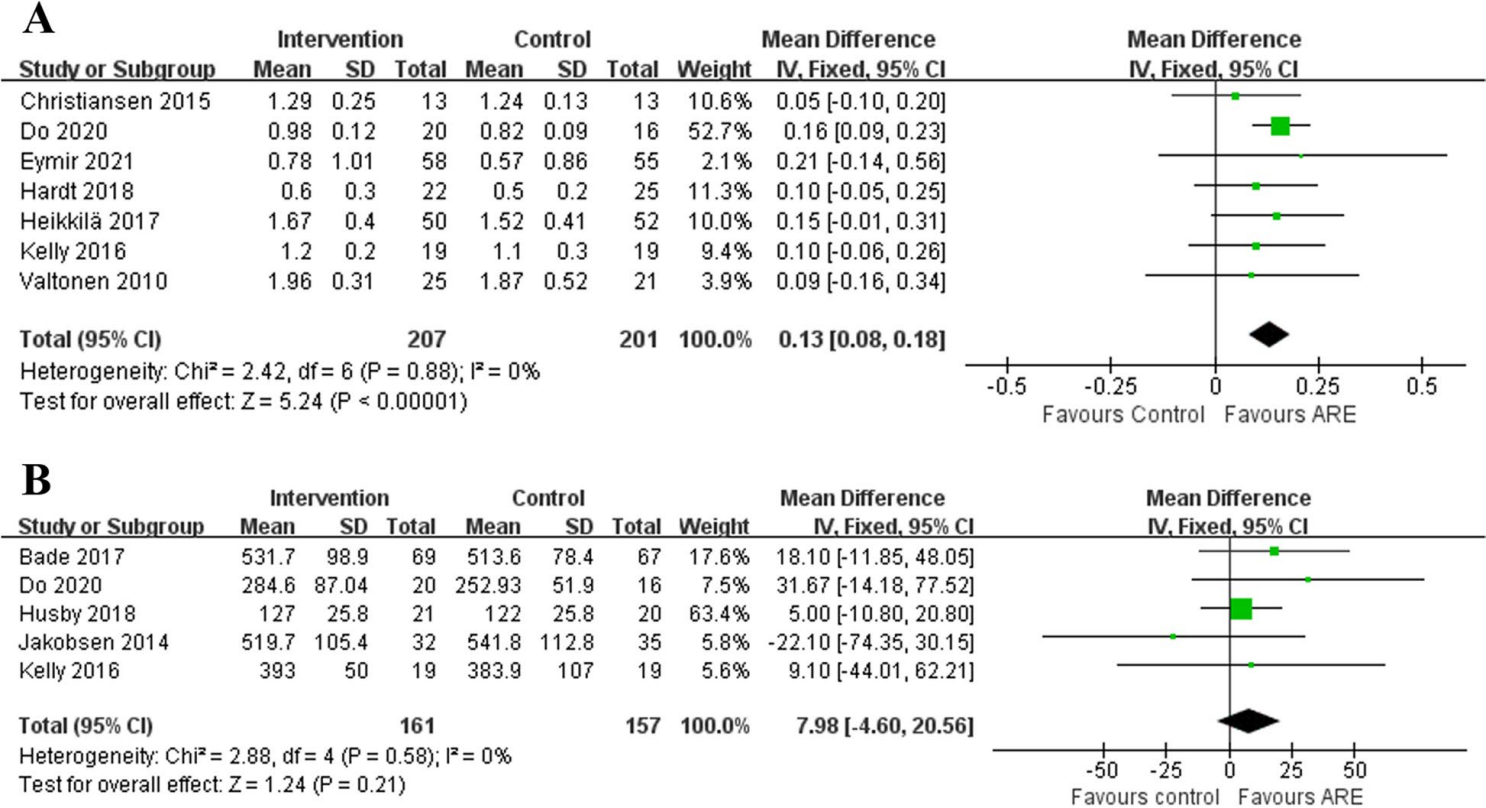
Forest plot of mobility (**A** MWS and **B** 6MWT)

Ten studies reported a knee mobility outcome using the MWS [13, 15, 21, 24, 26, 27, 30] or 6MWT [13, 24, 25, 28, 29]. The outcome from the pooled analysis of seven studies with 408 patients showed that patients in the ARE group had a significantly improved MWS compared to those in the CON group (MD 0.13, 95%CI 0.08–0.18, *P* < 0.00001; Fig. 3A), and significant heterogeneity was not observed (*P* = 0.88, I^2^ = 0%). Moreover, pooled estimates from five studies with 318 patients suggested there was little or no difference between the two groups for 6MWT (MD 7.98, 95%CI -4.60–20.56, *P* = 0.21; Fig. 3B), and no statistical heterogeneity was discovered (*P* = 0.58, I^2^ = 0%).

#### Function (TUG and SCT)

**Fig. 4:**
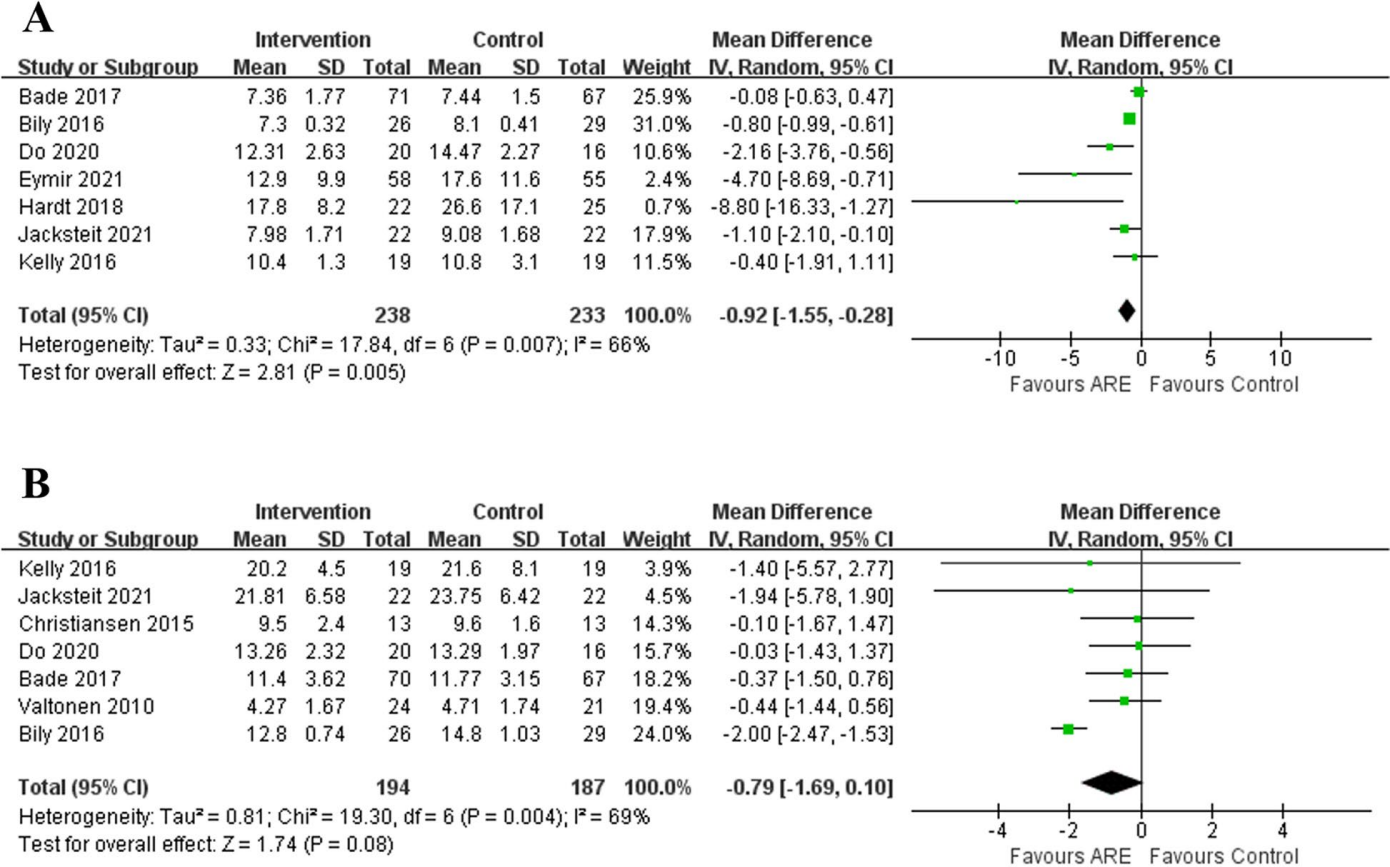
Forest plot of function (**A** TUG and **B** SCT)

The knee joint function was evaluated using TUG and SCT. Seven trials [13, 15, 16, 23–25, 27] including 471 patients compared the TUG between the ARE and CON. The random-effects model was used instead of a fixed-effects model due to the high heterogeneity (*P* = 0.007, I^2^ = 66%) of the combined TUG. The combined TUG was better in the ARE group than in the CON group, and the difference between the groups reached statistical significance (MD -0.92, 95%CI -1.55– -0.28, *P* = 0.005; Fig. 4A). Furthermore, SCT involving 381 patients was assessed in seven studies [13, 16, 21, 23–25, 30]. The meta-analysis result was heterogeneous (*P* = 0.004, I^2^ = 69%), so the random-effects model was used for further analysis. The results showed that the two groups had similar SCT (MD -0.79, 95%CI -1.69–0.10, *P* = 0.08; Fig. 4B).

#### Knee power (KEP and KFP)

**Fig. 5:**
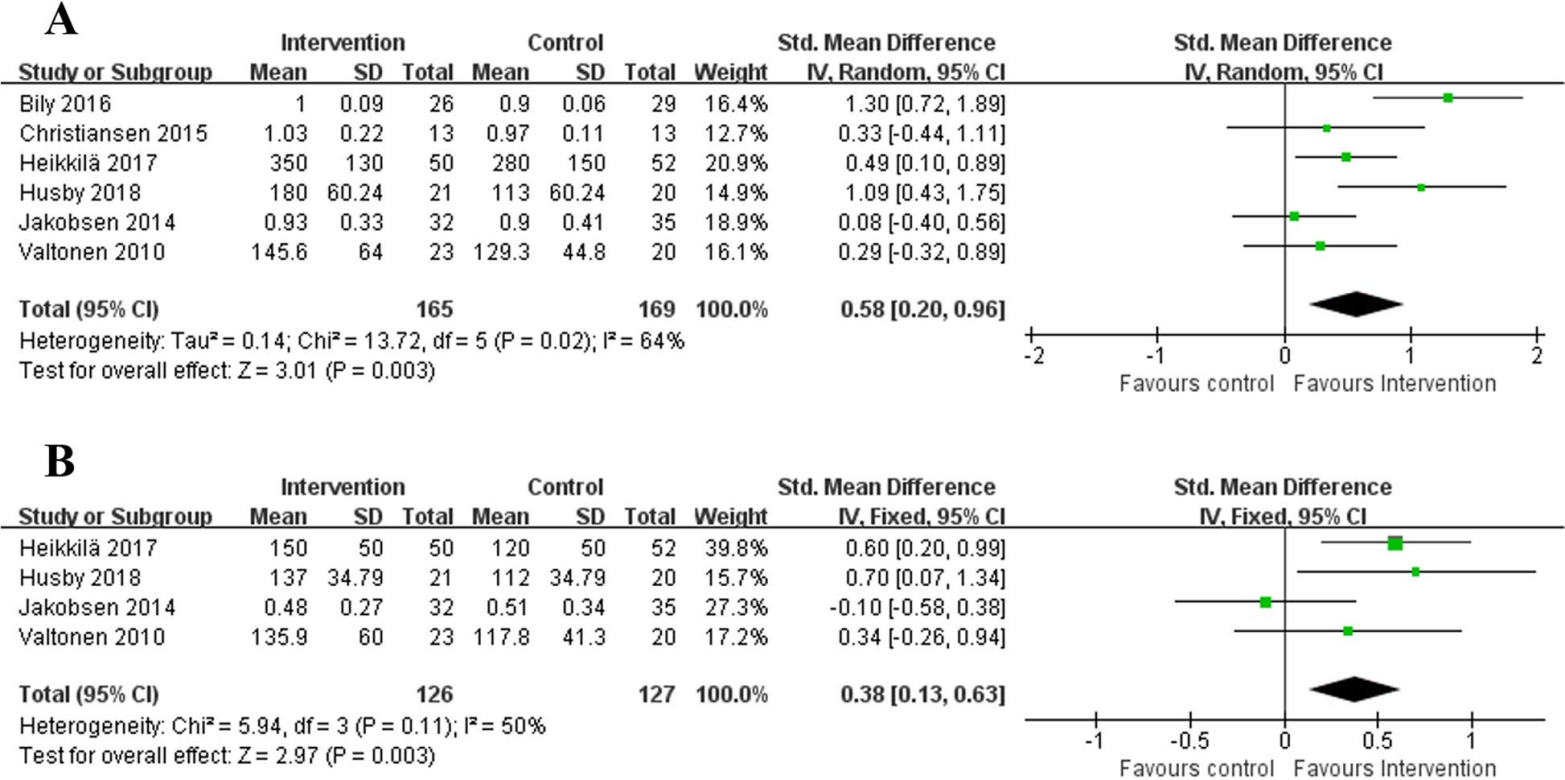
Forest plot of knee power (**A** KEP and **B** KFP)

The KEP data suitable for meta-analysis were available from six studies [21, 23, 26, 28–30] with 334 participants and for KFP from four study [21, 26, 28, 29] with 253 participants. Greater heterogeneity was found across studies reporting KEP(*P* = 0.02, I^2^ = 64%), so the random effects model was used for further analysis. The results showed that ARE significantly improved KEP (SMD 0.58, 95%CI 0.20–0.96, *P* = 0.003; Fig. 5A), but no source of heterogeneity was found. Similarly, Significant higher KFP was identified for patients with ARE after TKA in comparison to those without intervention(SMD 0.38, 95%CI 0.13– 0.63, *P* = 0.003; Fig. 5B). The between-study heterogeneity was not statistically obvious (*P* = 0.11, I^2^ = 50%), and fixed effect model was applied to assess the effect sizes.

### Secondary outcomes

#### ROM

**Fig. 6:**
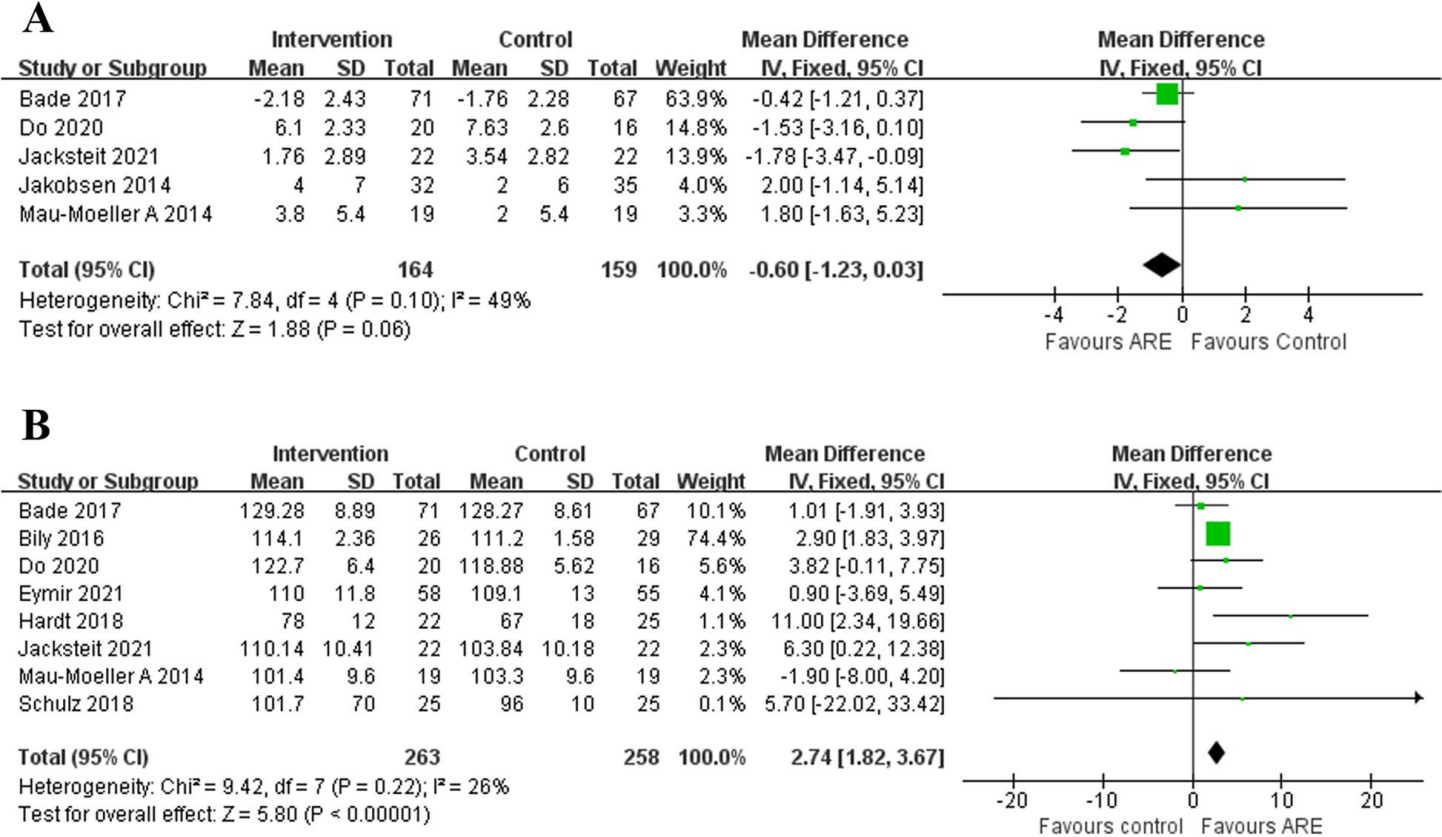
Forest plot of knee ROM (**A** extension and **B** flexion)

Our review found a total of nine relevant studies, including eight studies [12, 13, 15, 16, 22, 23, 25, 27] involving flexion range angle and five studies [13, 16, 22, 25, 29] involving extension range angle. Fixed effect model was used to analyze the pooled data since there was not heterogeneity. As seen from Fig. 6, the difference in extension range angle did not reach statistical significance (*P* = 0.1, I^2^ = 49%, MD -0.60, 95%CI -1.23–0.03, *P* = 0.06; Fig. 6A), but the difference in flexion range angle did (*P* = 0.22, I^2^ = 26%, MD 2.74, 95%CI 1.82–3.67, *P* < 0.00001; Fig. 6B). Furthermore, the ARE group had a higher mean flexion range angle than the CON group (*P* < 0.00001).

### Pain (VAS)

**Fig. 7:**
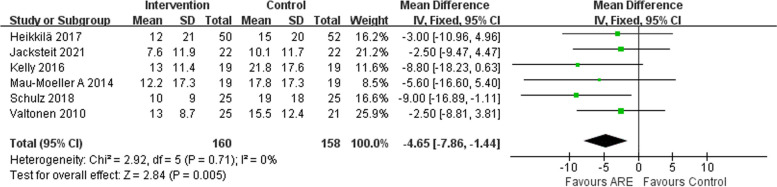
Forest plot of pain (VAS)

Data of VAS pain scores were available from six studies [12, 16, 21, 22, 24, 26]. Pooled estimates from six studies indicated that the ARE patients had a significant reduction on VAS (MD − 4.65, 95% CI − 7.86– -1.44, *p* = 0.005; Fig. 7), and significant heterogeneity was not observed (*P* = 0.71, I^2^ = 0%).

### Sensitivity analysis

A series of sensitivity analysis were conducted to assess the stability of synthesis results and to identify sources of heterogeneity by removing every single study and analyzing the effect on overall results. According to the analysis results, there was not a particularly influential study among all selected studies, apart from the impact of Jakobsen’s study [29] on KFP and Bily’s study [23] on KEP or SCT. When Jakobsen’s study was excluded, the heterogeneity decreased significantly from 50 to 0%. However, there was no significant change in the combined KFP (I^2^ = 0%, *P* = 0.69; SMD 0.56, 95%CI 0.26– 0.85, *P* = 0.0002). After eliminating Bily’s study, the pooled KEP and SCT results changed insignificantly (I^2^ = 36%, *P* = 0.18; SMD 0.42, 95%CI 0.18– 0.66, *P* = 0.0006; and I^2^ = 0%, *P* = 0.94; MD -0.35, 95%CI -0.95– 0.24, *P* = 0.25). Due to the small number of studies included, we did not undertake a publication bias assessment.

## Discussion

The current systematic review meta-analytically explored the literature to evaluate the effects of active resistance exercise after total knee arthroplasty. In the pooled study of around 880 participants from 14 randomized controlled trials, we chose 6MWT, MWS, SCT, TUG, ROM, knee extension/ flexion power and VAS to assess mobility, physical function, knee strength and pain intensity of patients post-operatively. The primary finding from our study consistently suggested that the lower-extremity ARE significantly improved knee flexion, knee extension and flexion power compared to conventional exercise.Similarly, significantly greater improvements were shown in mobility(MWS) and physical function(TUG) for the ARE group compared to the CON group, although no between-group significant differences were found for 6MWT and SCT. In terms of pain intensity, the results of this review showed that there was a significant improvement in the ARE group.

Previous studies have shown that surgery can damage the knee’s musculoskeletal structures and associated muscles, such as the quadriceps and hamstring muscles [41].Therefore, improving muscle strength was of great significance for postoperative rehabilitation. The lower-extremity ARE played an important role in preventing further loss of muscle strength and promoting muscle strength to return to normal after TKA. In recent years, some studies had found that preoperative strength training had multiple positive effects on rehabilitation after TKA, such as reducing pain, increasing muscle strength and range of motion,and promoting joint function recovery [42–44]. Furthermore, two systematic review and meta-analyses [38, 45] demonstrated preoperative strength training might be beneficial to early rehabilitation. As for the effect of postoperative resistance training, two systematic review and meta-analyses [46, 47] evaluating the clinical effectiveness of postoperative progressive resistance training for TKA had been published recently. Chen et al. [46]found that progressive resistance training early after THA or TKA did not differ significantly from conventional exercise in terms of functional capacity, muscle strength recovery and incidence of adverse events. What they found was in disagreement with the result of our meta-analysis which showed ARE was clinically significantly superior to CE in terms of knee flexion Angle, knee strength and pain. Our results showed that patients with postoperativeARE tended to present greater improvements, although statistically significant difference was not detected for the pooled extension range angle.The difference might be partially ascribed to small sample size and different score systems in the previous two systematic reviews, which might result in publication bias. Regarding the knee strength outcomes, A previous meta-analysis [47] showed that compared to a regular rehabilitation program, ARE significantly improved knee strength and was safe for TKA patients. This was consistent with our conclusion that knee strength and ROM consistently improved in patients undergoing TKA who were exposed to ARE postoperatively. Through ARE, muscle fibers were stimulated to proliferate and hypertrophy, thereby enhancing the strength and duration of muscle contraction. Resistance exercises effectively elicited strength gains, and both training volume and intensity were strongly associated with the level of physiological adaptations in healthy aging adults [48]. This was crucial for improving the support and motor function of the lower extremities after surgery. Moreover, ARE promoted neuromuscular adaptability. During the training process, the coordination of nerve impulse transmission and muscle response was enhanced, thereby improving the motor control ability and coordination of muscles, increasing the stability of the knee joint and its range of motion [49–51]. Considering the loss of muscle strength and muscle mass post-surgery immediately, ARE had been advocated to be initiated shortly following surgery [52]. For another, postoperative pain was one of the most important complications after TKA, which seriously affected the patient’s quality of life. Our study was the first meta-analysis that evaluated effects of PRE on pain intensity, and found significant advantage of ARE in improving pain after TKA. The possible explanation was that active movement and muscle activation during exercise would cause an increase in blood circulation, providing more efficient oxygen flow to the related muscles and joints. It would also stimulate the release of exercise-induced endogenous substances such as endorphins and enkephalins, ultimately reducing pain [53]. What's more, ARE might reduce inflammation period and support the healing process, which in turn might contribute to increased physical function. To sum up, the lower-limb ARE after TKA was through the combined effect of various physiological mechanisms, which required further research and confirmation in the future.

Regarding the mobility and physical function outcomes, there was no significant difference between the two groups with respect to the assessment of 6MWT and SCT. Nevertheless, combined measures of mobility (MWS) and physical function (TUG) exhibited significantly greater improvement for individuals undertaking ARE than those that completed CON rehabilitation. In the evidence based meta-analysis of Liu et al. [47], they found that there was significant advantage of ARE on 6MWT, but not on MWS. This might have resulted from the less precise timed-based metric test. Quadriceps strength was the strongest predictor of functional performance in patients following TKA. Higher quadriceps strength might positively impact functional performance of participants in the ARE group [14, 54]. Patients who received postoperative ARE showed better knee strength, ROM and pain relief after TKA.Therefore, it was not surprising that the participants in ARE group achieved the better 6MWT and SCT test compared to the participants in CON group. Similar trends were observed for MWS and TUG tests performance, although there were no statistical differences between the two groups. Although this study found that ARE was the key to restoring the mobility and improving physical function of TKA patients, considering that this effect was too small, the long-term evaluations were not conducted, and some other vital results (adverse event, length of hospital stay and medical expenses) had not been assessed, the clinical significance of the results might need to be interpreted more carefully.More attention should be given to the design of (unilateral) ARE interventions in terms of choice of exercises (optimizing the symmetry between the legs), intensity (as intensive as possible) and duration and frequency to optimise the effectiveness. Finally,future studies with larger sample sizes are needed to evaluate the influence of ARE on mobility and physical function in TKA patients.

### Limitations

We included only high-level studies in which treatment assignments were randomized, enhancing the strength of the conclusions that could be drawn from the findings. However, a number of potential limitations should be taken into account when interpreting our results. First of all, the program content, intensity and frequency of ARE and the follow-up period were not uniform across all studies, which might lead to the possibility of bias and heterogeneity. The authors have attempted to address this by assessing the I^2^ in every forest plot to ensure minimal heterogeneity affecting the study, which was found to not be statistically significant. Besides the clinical heterogeneity in exercise protocols, there was also a wide range of outcome measures used to evaluate function and knee power, further contributing to the high heterogeneity. Second, When extracting data, some literature were excluded due to lack of data and other reasons, leading to the defect of fewer included literatures. Moreover, the relatively limited quantity of included studies did not allow the evaluation of publication bias for the outcomes evaluated in the meta-analysis. Thirdly, the general lack of blinded assessors or participants may increase the risk of overestimation of the effects of the ARE interventions. Fourthly, the long-term outcomes for evaluation of ARE were lacking. Finally,we have not assessed the possibility of unpublished as well as non-English studies, so a publication bias may exist. For us, English reports are more likely to have better methodological quality than reports written in other languages [55]. Besides, Acquiring, translating and evaluating a large amount of non-English literature requires a significant investment of manpower, material resources and time, which may exceed the practical feasibility of the research. For grey literature, it may lack a rigorous peer review process, and the accuracy of the data and the scientific nature of the methods are difficult to guarantee, which may have an impact on the reliability of the analysis results.. Although the evidence may be imperfect, the results of this meta-analysis have implications for clinical practice, which can guide physicians and patients to make the appropriate choice for enhancing recovery after TKA.

## Conclusion

The present review and meta-analysis showed that patients undergoing TKA who received the lower extremity ARE showed better clinical effects in terms of pain relief, strength recovery, and knee ROM. Simultaneously, it might be beneficial to improve mobility and physical function of patients after TKA.Therefore, ARE is one of the options for rapid rehabilitation after TKA. Given these limitations, Sufficient, high-quality, prospective RCT with large samples are required to further elucidate the relationship between postoperative lower-limb ARE protocols and outcomes after TKA.

## Supplementary Information

Supplementary Material 1. Searching strategy for Each Database.

Supplementary Material 2. The summary table of the risk of bias.

Supplementary Material 3. GRADE evidence for the outcomes.

## Data Availability

The datasets used and/or analysed during the current study available from the corresponding author on reasonable request.

## Abbreviations

RCT: Randomized controlled trial
ARE: Active resistance exercise
MWS: Maximal walking speed
TKA: Total knee arthroplasty
HWS: Habitual walking speed
SMD: Standardized Mean Differences
KEP: Knee extension power
MD: Mean Differences
KFP: Knee flexion power
CE: Conventional exercise
6MWT: 6-Minute Walk Test
CI: Confidence intervals
SCT: Stair Climb Test
CPM: Continuous passive motion
TUG: Timed Up and Go
CON: Control
VAS: Visual Analogue Scale
ROM: Range of motion
