# Supplementary material for: Effects of lower-limb active resistance exercise on mobility, physical function, knee strength and pain intensity in patients with total knee arthroplasty: a systematic review and meta-analysis
Source: BMC Musculoskelet Disord. 2024 Sep 12;25:730. doi: 10.1186/s12891-024-07845-9 (PMC11395693; doi:10.1186/s12891-024-07845-9)
Supplement: Supplementary file 1 — Supplementary Material 1. Searching strategy for Each Database. [file 12891_2024_7845_MOESM1_ESM.docx]

**Pumbed search strategie: 576**

4# ("total knee arthroplasty"[Title/Abstract] OR "total knee replacement"[Title/Abstract]) AND ("rehabilitation"[Title/Abstract] OR "strengthening"[Title/Abstract] OR "resistance"[Title/Abstract] OR "training"[Title/Abstract] OR "exercise"[Title/Abstract] OR "active"[Title/Abstract] OR "progressive"[Title/Abstract]) AND (randomized controlled trial[Filter]) 576 9:54:38

3# ("total knee arthroplasty"[Title/Abstract] OR "total knee replacement"[Title/Abstract]) AND ("rehabilitation"[Title/Abstract] OR "strengthening"[Title/Abstract] OR "resistance"[Title/Abstract] OR "training"[Title/Abstract] OR "exercise"[Title/Abstract] OR "active"[Title/Abstract] OR "progressive"[Title/Abstract]) 3787 9:54:28

2# ((((((rehabilitation,[Title/Abstract]) OR (strengthening[Title/Abstract])) OR (resistance[Title/Abstract])) OR (training[Title/Abstract])) OR (exercise[Title/Abstract])) OR (active[Title/Abstract])) OR (progressive[Title/Abstract]) 3278370 9:51:45

1# (total knee arthroplasty [Title/Abstract]) OR (total knee replacement Title/Abstract])

31192 9:49:22

**Cochrane search strategy: 482**

#1 (total knee arthroplasty OR total knee replacement):kw AND (rehabilitation OR strengthening OR resistance training OR exercise OR active OR progressive):kw

**Embase search strategy: 335**

total knee arthroplasty and total knee replacement AND (rehabilitation OR strengthening OR resistance training OR exercise OR active OR progressive)
