# Supplementary material for: Effects of lower-limb active resistance exercise on mobility, physical function, knee strength and pain intensity in patients with total knee arthroplasty: a systematic review and meta-analysis
Source: BMC Musculoskelet Disord. 2024 Sep 12;25:730. doi: 10.1186/s12891-024-07845-9 (PMC11395693; doi:10.1186/s12891-024-07845-9)
Supplement: Supplementary file 3 — Supplementary Material 3. GRADE evidence for the outcomes. [file 12891_2024_7845_MOESM3_ESM.docx]

| **Active lower-limb resistance training compared to conventional exercise for Patients with Total Knee Replacement Arthroplasty** | | | | | | |
| --- | --- | --- | --- | --- | --- | --- |
| **Patient or population:** Patients with Total Knee Replacement Arthroplasty **Settings:**  **Intervention:** Active lower-limb resistance training **Comparison:** conventional exercise | | | | | | |
| **Outcomes** | **Illustrative comparative risks* (95% CI)** | | **Relative effect (95% CI)** | **No of Participants (studies)** | **Quality of the evidence (GRADE)** | **Comments** |
|  | Assumed risk | Corresponding risk |  |  |  |  |
|  | **Conventional exercise** | **Active lower-limb resistance training** |  |  |  |  |
| **6MWT** | The mean 6mwt in the control groups was **-** | The mean 6mwt in the intervention groups was **7.98 higher** (4.6 lower to 20.56 higher) |  | 318 (5 studies) | ⊕⊕⊝⊝ **low**^1,2,3,4^ |  |
| **MWS** | The mean mws in the control groups was **0** | The mean mws in the intervention groups was **0.13 higher** (0.08 to 0.18 higher) |  | 408 (7 studies) | ⊕⊕⊝⊝ **low**^1,2,3,5^ |  |
| **TUG** | The mean tug in the control groups was **0** | The mean tug in the intervention groups was **0.92 lower** (1.55 to 0.28 lower) |  | 471 (7 studies) | ⊕⊝⊝⊝ **very low**^1,2,3,4,6,7^ |  |
| **SCT** | The mean sct in the control groups was **0** | The mean sct in the intervention groups was **0.35 lower** (0.95 lower to 0.24 higher) |  | 326 (6 studies) | ⊕⊕⊝⊝ **low**^1,2,3,4^ |  |
| **KROM-flexion** | The mean krom-flexion in the control groups was **0** | The mean krom-flexion in the intervention groups was **2.74 higher** (1.82 to 3.67 higher) |  | 521 (8 studies) | ⊕⊕⊝⊝ **low**^1,2,3^ |  |
| **KROM-extension** | The mean krom-extension in the control groups was **0** | The mean krom-extension in the intervention groups was **0.6 lower** (1.23 lower to 0.03 higher) |  | 323 (5 studies) | ⊕⊕⊝⊝ **low**^1,3,4^ |  |
| **KEP** | The mean kep in the control groups was **0** | The mean kep in the intervention groups was **0.58 standard deviations higher** (0.2 to 0.96 higher) |  | 334 (6 studies) | ⊕⊝⊝⊝ **very low**^1,2,3,6,7^ | SMD 0.58 (0.2 to 0.96) |
| **KFP** | The mean kfp in the control groups was **0** | The mean kfp in the intervention groups was **0.38 standard deviations higher** (0.13 to 0.63 higher) |  | 253 (4 studies) | ⊕⊕⊝⊝ **low**^1,3^ | SMD 0.38 (0.13 to 0.63) |
| **Pain** | The mean pain in the control groups was **0** | The mean pain in the intervention groups was **4.65 lower** (7.86 to 1.44 lower) |  | 318 (6 studies) | ⊕⊕⊝⊝ **low**^1,3^ |  |
| *The basis for the **assumed risk** (e.g. the median control group risk across studies) is provided in footnotes. The **corresponding risk** (and its 95% confidence interval) is based on the assumed risk in the comparison group and the **relative effect** of the intervention (and its 95% CI).  **CI:** Confidence interval; | | | | | | |
| GRADE Working Group grades of evidence **High quality:** Further research is very unlikely to change our confidence in the estimate of effect.  **Moderate quality:** Further research is likely to have an important impact on our confidence in the estimate of effect and may change the estimate. **Low quality:** Further research is very likely to have an important impact on our confidence in the estimate of effect and is likely to change the estimate. **Very low quality:** We are very uncertain about the estimate. | | | | | | |
| ^1^ unblinding of participants and personnel ^2^ Intention-to-treat analysis was not applied for all outcome measures in some studies. ^3^ The sample size is small ^4^ The confidence interval is wide ^5^ No explanation was provided ^6^ The data is considered to have substantial heterogeneity(I2 >50%) ^7^ Publication bias may exist in the results | | | | | | |
